# Editorial: Near-infrared fluorescence guided surgery: State of the evidence from a health technology assessment perspective

**DOI:** 10.3389/fsurg.2023.1176124

**Published:** 2023-03-17

**Authors:** Luca Bertolaccini, Oriana Ciani

**Affiliations:** ^1^ Department of Thoracic Surgery, IEO, European Institute of Oncology IRCCS, Milan, Italy; ^2^ Centre for Research on Health and Social Care Management (CERGAS), SDA Bocconi School of Management, Milan, Italy

**Keywords:** near-infrared fluorescence guided surgery, lung cancer, thoracic surgery, health technology assessment, VATS

**Editorial on the Research Topic** Near-infrared fluorescence guided surgery: State of the evidence from a health technology assessment perspective

Indocyanine green is a fluorescent dye used in various medical applications, including thoracic surgery. Indocyanine green-based near-infrared fluorescence imaging can provide real-time visualization of the vascular and lymphatic structures, aiding in the identification of critical anatomical structures during thoracic surgeries (1–4).

Some potential applications of indocyanine green-based near-infrared fluorescence imaging in thoracic surgery include:

1. Pulmonary segmentectomy.
  Near-infrared fluorescence imaging using Indocyanine green can aid in the identification of segmental bronchi and vessels, which can help in the precise identification and preservation of pulmonary segments during segmentectomy. Due to the development of imaging technologies, there has been an increase in the discovery of small, nonpalpable lung nodules previously undetected. Long-awaited randomized controlled experiment JCOG0802 demonstrated that segmentectomy is superior to lobectomy for lung tumors 2 cm in size. Consequently, it is reasonable to predict that segmentectomies will replace lobectomies for stage IA illness (5). Before segmentectomies can be regarded as the standard of care for this subset of patients and widely adopted by thoracic surgeons, at least three pillars must be established: the non-inferiority of segmentectomy to lobectomy in terms of oncologic efficacy, the technical feasibility, and a good cost-effectiveness profile vis-à-vis the standard approach.
  Consequently, the tumor’s location is essential for thoracic surgeons undergoing segmentectomies. When nodules are discovered in peripheral segments or intersegmental planes, it is essential to examine the tumor’s location and segmental region to give radical surgical margins. Precise knowledge of the nodule and intersegmental plane is necessary to accomplish a complex segmentectomy (6).
2. Mediastinal lymph node dissection.
  Indocyanine green-based near-infrared fluorescence imaging can identify the lymphatic drainage of tumors in the mediastinum, allowing for more accurate identification and dissection of lymph nodes during surgery. After peritumoral injection, it can help identify sentinel lymph nodes and observe the lymphatic drainage path during lung resections, allowing for correct lymphadenectomy and N staging, or it can aid in the treatment of problems such as chylorrhea by locating the thoracic duct. Near-infrared fluorescence fluorescence-guided sentinel lymph node mapping can correctly identify the sentinel lymph node, allowing the discovery of micrometastases, providing accurate staging, and possibly improved survival. Using the so-called improved permeability and retention, a passive targeting effect 24 h after the injection of a hefty dose of near-infrared fluorescence can visualize even small pulmonary nodules, filling the vacuum following the loss of tactile input during VATS. Other fluorescent dyes are more specific for adenocarcinomas, although their usage is uncommon (7).
3. Identification of pulmonary nodules.
  Near-infrared fluorescence imaging using Indocyanine green can aid in the identification of small pulmonary nodules, which can be difficult to visualize with conventional imaging techniques. The indications and complications associated with fluorescence-navigated thoracoscopy have been summarized in a recent consensus study. Specifically, it may be a suitable method for identifying pulmonary nodules, intersegmental planes during thoracoscopic segmentectomy, and the surgical margin after excision. In contrast, given the low-quality evidence for the detection of sentinel lymph nodes, additional research is required to evaluate its role in this context. In addition, the panel of experts felt that fluorescence-guided thoracoscopic surgery could become a standard procedure for treating pulmonary lesions (8).
4. Assessment of pulmonary perfusion.
  Near-infrared fluorescence imaging using Indocyanine green can provide real-time visualization of pulmonary perfusion, which can help evaluate lung function and identify areas of impaired perfusion (1–4).
5. Thoracoscopic sympathectomy.
  Indocyanine green-based near-infrared fluorescence imaging can aid in the identification of the sympathetic chain during thoracoscopic sympathectomy, which can help reduce the risk of nerve injury (1–4).

Overall, Indocyanine green-based near-infrared fluorescence imaging has the potential to enhance the precision and safety of thoracic surgery by providing real-time visualization of anatomical structures and improving the accuracy of surgical interventions. Evidence for the safety and effectiveness of fluorescent imaging in thoracic surgery appears convincing, representing an excellent approach with minimal complications and contraindications. However, given the fundamental importance of a multidimensional assessment of health innovation to guide its diffusion and uptake across healthcare systems, additional research to clarify the managerial, economic, and cost-effectiveness impact of these options is still needed.

In this Research Topic for the *Frontiers in Surgery* Journal (Table 1), we present state-of-the-art on the clinical and patient-reported outcomes and organizational consequences of future improvements in fluorescence-guided surgery.

**Table 1:** Articles published within the research topic of *Frontiers in surgery*: near-infrared fluorescence guided surgery: state of the evidence from a health technology assessment perspective.

Article type	Authors	Title	DOI
Case report	Mi et al.	The second near-infrared window indocyanine green angiography in giant mediastinal tumor resection	https://doi.org/10.3389/fsurg.2022.852372
Case report	Londero et al.	Fluorescence-guided identification of the thoracic duct by VATS for treatment of postoperative chylothorax: a short case series	https://doi.org/10.3389/fsurg.2022.912351
Case report	Voulaz et al.	Preoperative CT-guided near-infrared dye marking for thoracoscopic resection of pulmonary nodules: a case report	https://doi.org/10.3389/fsurg.2022.919227
Mini review	Géczi T et al.	Near-infrared fluorescence guided surgery: state of the evidence from a health technology assessment perspective	https://doi.org/10.3389/fsurg.2022.919739
Original research	Chan et al.	Robotic assisted-bronchoscopy with cone-beam CT ICG dye marking for lung nodule localization: experience beyond USA	https://doi.org/10.3389/fsurg.2022.943531
Mini review	Tajè et al.	Fluorescence-guided lung nodule identification during minimally invasive lung resections	https://doi.org/10.3389/fsurg.2022.943829
Mini review	Tamburini et al.	Application of indocyanine green enhanced fluorescence in esophageal surgery: a mini review	https://doi.org/10.3389/fsurg.2022.961856
Systematic review	Gkikas et al.	How effective is indocyanine green (ICG) in localization of malignant pulmonary nodules? A systematic review and meta-analysis	https://doi.org/10.3389/fsurg.2022.967897
